# Enhance the Quality of Crowdsensing for Fine-Grained Urban Environment Monitoring via Data Correlation

**DOI:** 10.3390/s17010088

**Published:** 2017-01-04

**Authors:** Xu Kang, Liang Liu, Huadong Ma

**Affiliations:** Beijing Key Laboratory of Intelligent Telecommunications Software and Multimedia, Beijing University of Posts and Telecommunications, Beijing 100876, China; kangxu88@bupt.edu.cn (X.K.); mhd@bupt.edu.cn (H.M.)

**Keywords:** crowdsensing, urban sensing, environment monitoring

## Abstract

Monitoring the status of urban environments, which provides fundamental information for a city, yields crucial insights into various fields of urban research. Recently, with the popularity of smartphones and vehicles equipped with onboard sensors, a people-centric scheme, namely “crowdsensing”, for city-scale environment monitoring is emerging. This paper proposes a data correlation based crowdsensing approach for fine-grained urban environment monitoring. To demonstrate urban status, we generate sensing images via crowdsensing network, and then enhance the quality of sensing images via data correlation. Specifically, to achieve a higher quality of sensing images, we not only utilize temporal correlation of mobile sensing nodes but also fuse the sensory data with correlated environment data by introducing a collective tensor decomposition approach. Finally, we conduct a series of numerical simulations and a real dataset based case study. The results validate that our approach outperforms the traditional spatial interpolation-based method.

## 1. Introduction

Rapid urbanization makes the issue of urban environment more serious than ever before in major cities, especially for developing countries. Urban environment monitoring, which provides crucial information for scientific city management, is of great importance for solving urban environmental problems. *Urban Remote Sensing* (URS) [1] and specialized *Wireless Sensor Network* (WSN) [2,3] are commonly used ways for acquiring a wide range of environmental monitoring data. However, the principle of URS limits the precision for fine-grained urban sensing. While for WSN, the sensing nodes always face the limited energy [4] and instability [5] problem, which restricts the scalability and utility of WSN for urban sensing. Therefore, it is very necessary to develop innovative technologies that could precisely and ubiquitously sense urban dynamics.

**Figure 1:**
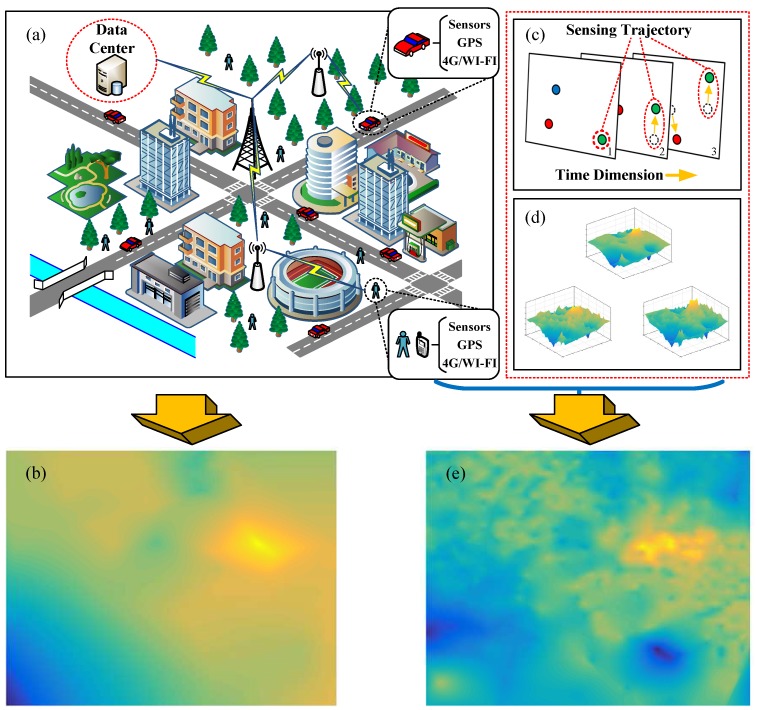
Crowdsensing networks based urban environment monitoring. (**a**) The crowdsensing networks, consisting of vast smartphones/vehicles, work as an urban camera; (**b**) Sensing image of target phenomenon generated by the crowdsensing network via interpolation method; (**c**) Sensing trajectories; (**d**) Correlated signals; (**e**) Sensing image after using enhanced crowdsensing approach.

Recent years, with the popularity of smartphones/vehicles equipped with onboard sensors and the development of wireless networks (such as 4G/Wi-Fi network), a people-centric sensing mode is emerging [6,7]. Researchers characterize this sensing mode as *crowdsensing* [8]. For urban monitoring with a crowdsensing network, mobile smartphones/vehicles equipped with sensing modules in a region send their measurements (sensory data) to a data center via wireless network, then the data center aggregates these measurements to estimate the phenomenon values of all points in the region. Similar to digital images, the distribution status of environment phenomenon (such as PM2.5 concentration, CO${}_{2}$ concentration) over a region can be expressed as a two-dimensional (2D) signal. To illustrate fine-grained urban status, the crowdsensing network utilize sensory data to generate a *sensing image* according to spatial interpolation methods [9]. As shown in Figure 1a,b, the crowdsensing network works like an “urban camera”, which could capture the urban status.

Generally, the geographical positions of crowdsensing participants are dynamic and uneven distributed in an urban area, which makes the quality of sensing image varies by time and locations significantly. In our previous work [10], we first defined a metric – *urban resolution* – for measuring the quality of sensing image. Learning from the concept of resolution in conventional image system, higher/lower urban resolution of sensing image means more/less details the sensing image holds. In fact, this metric is a measurement for evaluating the sensing ability (sensitivity) of crowdsensing network. Furthermore, we also revealed a linear relationship between urban resolution *r* and the number of crowdsensing participants *s* from statistical perspective.

Intuitively, huge number of crowdsensing participants will provide a guarantee of high urban resolution. In practice, regardless of the number of crowdsensing participants, the spatial distribution of participants is uneven, i.e., there exist some hot zones and blank zones simultaneously in the urban area. This inhomogeneity is determined by the underlying human mobility features, which are summarized from real mobility traces [11]. Based on our observation, the emergence of blank regions will obviously reduce the sensing ability of the crowdsensing network. Thus, how to increase the sensing ability for blank zones becomes the main challenge.

Recently, many researchers devote to breaking the primary bottleneck of crowdsensing, i.e., sensory data sparsity, which is mainly caused by limited number or uneven distribution of crowdsensing participants [12,13]. A powerful and generic technique, *Compressive Sensing* (CS), is extensively used for inferring the missing sensory data. By far, the application of CS has led to significant advances in reconstructing network traffic [14], improving urban traffic sensing [15] and refining localization [16], etc. Among these works, the CS-based methods share the same prerequisite that the sensory data should have inherent structure and can be sparsely represented. However, the CS-based method cannot achieve good performance for some common types of crowdsensing data (e.g., environment data), because the real environment data rarely satisfy the aforementioned technical conditions [17,18,19]. Fortunately, several works [17,20,21] have revealed both the temporal-spatial correlation and category correlation of environmental phenomenons through extensive real dataset analysis. These works motivate us to enhance the estimation accuracy of missing values for environment crowdsensing via a data correlation based approach. Here, the data correlation mainly refers to the following two aspects:

- Temporal correlation of sensory data. Researchers of [17] revealed the pervasive existence of time stability feature among environmental phenomenons, such as temperature, humidity and light. This feature indicates that most environmental phenomenons will not change dramatically and maintain stable for a while. On the other hand, the frequency of participants sending their measurements is much higher than that of environment changing. Utilizing temporal correlation is to leverage measurement data in a correlated time period rather than a moment. Due to the dynamic feature of crowdsensing participants, a discrete participant at a moment is converted into a sensing trajectory in correlated time period (see Figure 1c), which could decrease the area of blank zones.
- Category correlation of sensory data. Many existing researches [20,21,22] show the strong correlation among some categories of sensory data (see Figure 1d). Taking air quality data for example, three mainly concerned atmospheric pollutants, the concentration of PM2.5, PM10, and NO${}_{2}$, have clear correlation. Therefore, if there exist some correlated sensory data in blank zones, then the correlated information is able to recover the target environmental phenomenon.

In this paper, we first express a sensing image as the form of matrix (discrete signal matrix) and utilize a Delaunay triangulation based interpolation to complete the matrix as a baseline. Then, we build a three-dimension tensor (each dimension denotes region, signal category and time slot respectively) to model measurements collected by participants, and summarize a signal correlation matrix to measure the correlation between different categories of environment phenomenons. After that, we propose a collaborative tensor decomposition approach to supplement the missing entries on the basis of temporal and category correlation of sensory data. In order to verify the enhancement of crowdsensing ability, we utilize the *Self-similar Least Action Walk* (SLAW) model [11], a commonly used human/vehicles mobility model to generate trajectories of crowdsensing participants. Then, we reconstruct a CO${}_{2}$ concentration signal as the ground truth of target environment phenomenon, and further generate correlated signals through liner/non-liner functions. By conducting a series of simulations, we verify the promotion of crowdsensing ability. In addition, we also utilize real urban air pollutants data to examine our enhanced crowdsensing approach.

The rest of the paper is organized as follows. Section 2 presents the method for generating sensing image and defines crowdsensing resolution. Section 3 proposes the enhanced crowdsensing approach. Section 4 utilizes numerical simulations to investigate the resolution promotion of enhanced crowdsensing approach. Section 5 illustrates a case study by utilizing real dataset of urban air pollutants. The paper concludes in Section 6.

## 2. Urban Monitoring via Crowdsensing and Sensing Restriction of Crowdsensing

In this section, we first briefly introduce the process of generating sensing image via crowdsensing networks. Then, we give the definition of crowdsensing resolution, which is utilized to quantize the sensing ability of crowdsensing networks. At last, we give the linear restriction of crowdsensing resolution, which limits the sensing ability of crowdsensing networks.

### 2.1. Generation of Sensing Image via Crowdsensing Network

For crowdsensing based environmental monitoring applications, the participants, i.e., mobile smartphones/vehicles equipped with sensing modules, are the sensing nodes. We define this kind of sensing nodes as *crowdsensing node*.

**Definition** **1.** Crowdsensing Node. A smartphone/vehicle with sensing and communication abilities is called a crowdsensing node, denoted by $u_{i}$, if the smartphone/vehicle senses the natural phenomenon. The value of $u_{i}$ is represented as $u_{i}\left(x_{i},y_{i},v_{i},t_{i}\right)$ where $\left(x_{i},y_{i}\right)$, $v_{i}$ and $t_{i}$ denote the position, measurement and the corresponding time of $u_{i}$, respectively.

**Figure 2:**
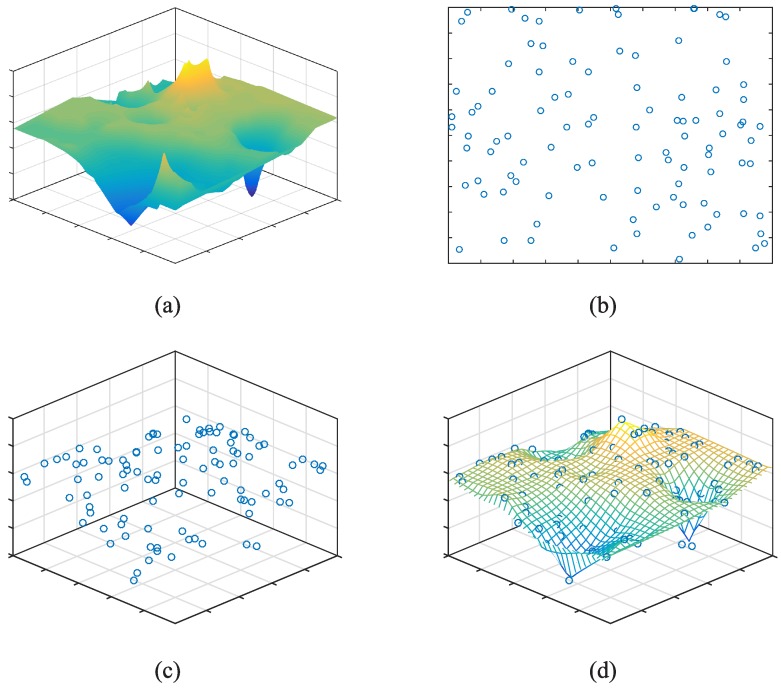
The process of generating sensing image via crowdsensing. (**a**) The 2D signal of ${\mathrm{CO}}_{2}$ concentration; (**b**) The distribution of 100 nodes in *R* with their sending time $t_{i}\in T$; (**c**) The sampling points of 2D signal; (**d**) Sensing image generated by *V*.

The natural phenomenon in a city is dynamic, while for a small time interval denoted by *T*, it can be expressed as a static two-dimensional (2D) signal. Mathematically, the signal is a function of two independent variables, i.e., spatial position (x,y) and the phenomenon value is v=F(x,y). Figure 2a shows a case of 2D continuous-space signal (distribution of ${\mathrm{CO}}_{2}$ concentration over a region) in *T*. Assume that the urban region is a square *R* and *s* crowdsensing nodes are dynamically distributed in *R*. Figure 2b illustrates the distribution of 100 crowdsensing nodes at time $t_{i}\in T$. Let $U=\{u_{i},1\leq i\leq s\}$ be the set of crowdsensing nodes in *R*, and $V=\{v_{i},1\leq i\leq s\}$ be the set of measurements. In other words, *V* is the set of sampling points of F(x,y) (see Figure 2c). Generation of sensing image, is to estimate the phenomenon values of all points in *R* via *V* (see Figure 2d).

**Figure 3:**
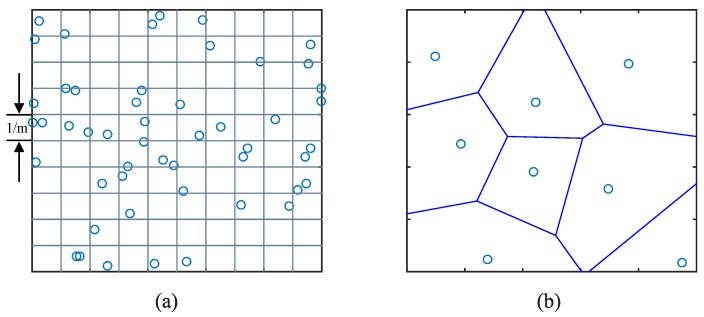
(**a**) Division of unit square *R* by m×m uniform grids; (**b**) A voronoi diagram based on the locations of crowdsensing nodes.

In order to implement this estimation, we transform F(x,y) from 2D continuous-space signal to a 2D discrete-space signal by dividing the unite square *R* into m×m grids (see Figure 3a). Each grid has a uniform signal value. Then, the 2D signal could be expressed by a matrix $Z_{m\times m}$ for time period *T*. Because of the uneven distribution of crowdsensing nodes, which is determined by human mobility, there exists three situations for a given grid $g_{i,j}$:

- There is only one crowdsensing node in $g_{i,j}$. For this case, the corresponding entry $z_{i,j}$ in matrix Z is equal to the sensory data provided by the only crowdsensing node.
- There are more than one crowdsensing nodes in $g_{i,j}$. The corresponding $z_{i,j}$ is calculated by a weight sum of all the sensory data generated in $g_{i,j}$. Considering the distribution of crowdsensing nodes in one grid, we build a voronoi diagram according to the locations of crowdsensing nodes (see Figure 3b), and then calculate the weight sum of sensory data where the area of the divided polygons are weights of sensory data.
- There is no crowdsensing node in $g_{i,j}$. The corresponding $z_{i,j}$ is set to null.

A commonly used way for filling the null entries in Z is spatial interpolation method. More details about spatial interpolation can be found in [9]. Based on the interpolation method, we give the definition of sensing image as follows:

**Definition** **2.** *Sensing Image. For a given time interval T, the sensing image generated by the set of crowdsensing nodes, U, is defined as the following 2D matrix:*

$$Z^{\prime }={\left[\begin{array}{cccc} z_{1,1(T)}^{\prime } & z_{1,2(T)}^{\prime } & \cdots & z_{1,m(T)}^{\prime }\\ z_{2,1(T)}^{\prime } & z_{2,2(T)}^{\prime } & \cdots & z_{2,m(T)}^{\prime }\\ \vdots & \vdots & \ddots & \vdots \\ z_{m,1(T)}^{\prime } & z_{m,2(T)}^{\prime } & \cdots & z_{m,m(T)}^{\prime } \end{array}\right]}_{m\times m,}$$

*where*

(1) $$z_{i,j(T)}^{\prime }=\left\{\begin{array}{c} \sum_{u_{k}\in U_{i,j}}v_{k}\frac{a_{k}}{g},\;\;\;\;\left|U_{i,j}\right|>0;\\ I_{n}\left(V^{\prime },g_{i,j}\right),\;\;\;\;\left|U_{i,j}\right|=0. \end{array}\right.$$

In Equation (1), $U_{i,j}$ denotes the set of crowdsensing nodes in grid $g_{i,j}$ within *T*. Besides, *g*, $a_{k}$ are the area of the whole grid $g_{i,j}$ and the polygon centered on $u_{k}$ in the corresponding voronoi diagram respectively. $I_{n}\left(\cdot ,\cdot \right)$ denotes the interpolation method and $V^{\prime }\overset{\Delta }{=}\left\{z_{i,j(T)}^{\prime }:\forall g_{i,j},\left|U_{i,j}\right|>0\right\}$.

### 2.2. Resolution of Crowdsensing

As mentioned in Section 1, to measure the ability of crowdsensing is to estimate the quality of sensing image, i.e., QoI. Firstly, we give the definition of QoI as follows:

**Definition** **3.** *Quality of sensing Image. The quality of a sensing image for a specific time interval T, is defined as the similarity between the sensing image $Z^{\prime }$ and the static 2D discrete-space raw signal Z of the target environment phenomenon. Correlation coefficient is used for measuring the similarity, i.e.,*

$$\begin{array}{c} C\left(Z^{\prime },Z\right)=\frac{\sum_{i=1}^{m}\sum_{j=1}^{m}\left(z_{i,j(T)}-{\bar{z}}_{i,j(T)}\right)\left(z_{i,j(T)}^{\prime }-{\bar{z}}_{i,j(T)}^{\prime }\right)}{\sqrt{\;\;\left(\;\sum_{i=1}^{m}\sum_{j=1}^{m}{\left(z_{i,j(T)}-{\bar{z}}_{i,j(T)}\right)}^{2}\;\right)\;\;\left(\;\sum_{i=1}^{m}\sum_{j=1}^{m}{\left(z_{i,j(T)}^{\prime }-{\bar{z}}_{i,j(T)}^{\prime }\right)}^{2}\;\right)}}. \end{array}$$

A large correlation value means a strong similarity between $Z^{\prime }$ and Z, i.e., the QoI of $Z^{\prime }$ is high. However, for most cases, the raw signal Z is unknown in advance. Therefore, users cannot calculate the QoI by using this equation directly. Then, we turn to use a new metric, *sensing image resolution* (i.e., urban resolution proposed in [10]), to measure the quality of sensing image $Z^{\prime }$.

In conventional image systems, *resolution*, defined as the number of gridded pixels, is a metric to evaluate the quality of image. Similarly, resolution can also be used to measure the quality of sensing image. However, the challenge is that the resolution of sensing image is not simply the crowdsensing nodes’ count. As the crowdsensing nodes are dispersedly and dynamically distributed and not the strictly gridded distribution.

Indirectly, if we could find out how many gridded sensing nodes are needed to generate a sensing image, denoted by $Z^{\prime \prime }$, which has the similar QoI with $Z^{\prime }$ generated by *s* crowdsensing nodes, then the number of gridded sensing nodes, n×n, can be regarded as the resolution of sensing image $Z^{\prime }$. On the other hand, the corresponding n×n is also an estimation for the sensing ability of crowdsensing network. Therefore, we redefine this metric as *crowdsensing resolution*.

**Definition** **4.** *Resolution of Crowdsensing. The resolution, denoted by r, of a crowdsensing network with s sensing nodes is defined as $n_{l}\times n_{l}$ where*

(2) $$n_{l}=arg\;\underset{n}{max}\;C\left(Z^{\prime },Z^{\prime \prime }\right).$$

In Equation (2), $Z^{\prime }$ denotes the sensing image generated by the crowdsensing network, and $Z^{\prime \prime }$ denotes a sensing image generated by n×n gridded sensing nodes.

Obviously, when $n_{l}$ becomes large enough, $Z^{\prime \prime }$ could approximately equal to Z, i.e., $C\left(Z^{\prime \prime },Z\right)\approx 1$. That is to say, the crowdsensing network is with high sensing ability.

### 2.3. Linear Restriction

In paper [10], we utilized three 2D signals with different variation degrees to analyze the relationship between crowdsensing resolution *r* and sensing nodes number *s* via Monte Carlo simulations. An approximately linear growth relationship between *r* and *s* is revealed.

For *s* crowdsensing nodes in an unit area, the relationship between $n_{l}$ and *s* follows:

$$n_{l}=\alpha \sqrt{s},$$

where the slope *α* is with the reference value in $\left[0.5,0.6\right]$.

Therefore, given the number of crowdsensing participants *s*, we can easily infer the resolution *r* of the crowdsensing network via the linear relationship. On the other hand, the linear relationship performs as a restriction, which limits the sensing ability that a crowdsensing network could achieve according to the scale of crowdsensing participants.

## 3. Enhanced Crowdsensing Approach

To enhance the crowdsensing ability and break the linear restriction, we utilize both signal and temporal correlation of sensory data to generate sensing images more precisely. Firstly, we introduce how to model the sensing data acquired by crowdsensing participants. Then, we propose a collaborative tensor decomposition approach to infer the missing items of target signal through signals’ correlation. At last, we illustrate the correlated time slots combination approach to further infer the rest missing items via temporal correlation of environmental signal.

### 3.1. Data Modeling

**Figure 4:**
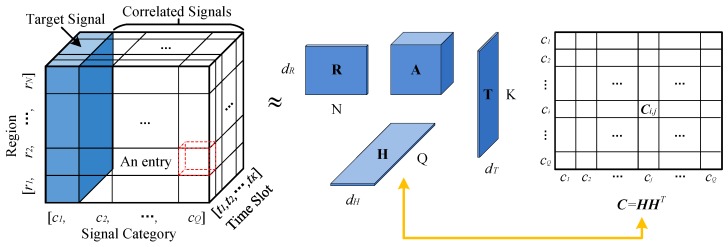
Collaborative tensor decomposition.

As shown in the left part of Figure 4, we model the sensory and correlated sensory data using a tensor, $S\in R^{N\times Q\times K}$, with three dimensions:

- *Region dimension*, $r=\left[r_{1},r_{2},\cdots ,r_{N}\right]$, denotes *N* regions which are transferred from m×m grids, one region per grid, $g_{i,j}=r_{im+j}$.
- *Category dimension*, $c=\left[c_{1},c_{2},\cdots ,c_{Q}\right]$, denotes *Q* signal categorise, where $c_{1}$ is the target signal and the others are correlated signals.
- *Time dimension*, $t=\left[t_{1},t_{2},\cdots ,t_{K}\right]$, denotes *K* time slots. Here, we divide the monitoring time period into *K* time slots and the span of each time slot is decided by the sending intervals of crowdsensing nodes.

An entry $S_{i,j,k}$ stores the sensory data, which is acquired in region $r_{i}$ at time slot $t_{k}$ with signal category of $c_{j}$. Likewise, for the process of mapping data to the tensor S, there also exists three situations as mentioned in Section 2.1 for a specific region and we adopt the same strategy to calculate the sensory data.

As tensor S is sparse, we try to “borrow” more information from correlation between different categories of signals for inferring the missing entries. Although tensor S can capture the correlation between different categories of signals to some context, a specialized matrix can further intensify the correlation. As shown in the right part of Figure 4, we formulate a matrix $C\in R^{Q\times Q}$ to model the signals’ correlation, where an entry $C_{i,j}$ denotes the correlation between signal $c_{i}$ and $c_{j}$. The correlation is quantized by:

(3) $$\begin{array}{c} C_{i,j}=\frac{\sum_{t}\sum_{r}\left(S_{r,i,t}-{\bar{S}}_{r,i,t}\right)\left(S_{r,j,t}-{\bar{S}}_{r,j,t}\right)}{\sqrt{\left(\sum_{t}\sum_{r}{\left(S_{r,i,t}-{\bar{S}}_{r,i,t}\right)}^{2}\right)\left(\sum_{t}\sum_{r}{\left(S_{r,j,t}-{\bar{S}}_{r,j,t}\right)}^{2}\right)}}, \end{array}$$

where $\sum_{t}$ is $\sum_{t=1}^{K}$ and $\sum_{r}$ is $\sum_{r=1,S_{r,i,t}\neq null,S_{r,j,t}\neq null}^{N}$.

After data modeling, we get a sparse tensor $S_{N\times Q\times K}$ and a signal correlation matrix, $C_{Q\times Q}$.

### 3.2. Collaborative Tensor Decomposition

To achieve a higher accuracy of filling the missing entries in tensor S, we exploit the collaborative tensor decomposition method. As shown in the middle part of Figure 4, tensor S is transformed into tucker decomposition model [23], i.e., the multiplication of a core tensor $A\in R^{d_{R}\times d_{H}\times d_{T}}$ with three matrices, $R\in R^{N\times d_{R}},H\in R^{Q\times d_{H}},T\in R^{K\times d_{T}}$. Here, $d_{R},d_{H},d_{T}$ are very small, denoting the number of latent factors. Moreover, the signal correlation matrix C is decomposed as the self product of matrix $H_{Q\times d_{H}}$, where $d_{H}<Q$, by low-rank approximation. By requiring them share the same low rank matrix $H_{Q\times d_{H}}$, we could propagate the information between tensor S and matrix C. Then, we define a loss function to quantize the error of collaborative tensor decomposition as:

(4) $$\begin{array}{cc} \Gamma \left(A,R,H,T\right)= & \frac{1}{2}{∥I\circ \left(S-A\times_{R}R\times_{H}H\times_{T}T\right)∥}^{2}+\frac{\lambda_{1}}{2}{∥C-HH^{T}∥}^{2}\\ & +\frac{\lambda_{2}}{2}\left({∥A∥}^{2}+{∥R∥}^{2}+{∥H∥}^{2}+{∥T∥}^{2}\right), \end{array}$$

where $∥\cdot ∥$ denotes the Frobenius norm, I is an indicating tensor, and the operator “∘” denotes the entry-wise product. The entry $I_{i,j,k}=0$ if $S_{i,j,k}$ is missing, $I_{i,j,k}=1$ otherwise. In Equation (4), ${∥I\circ \left(S-A\times_{R}R\times_{H}H\times_{T}T\right)∥}^{2}$ and ${∥C-HH^{T}∥}^{2}$ are to measure the errors of decomposing S and C, respectively, and the last part ${∥A∥}^{2}+{∥R∥}^{2}+{∥H∥}^{2}+{∥T∥}^{2}$ is regularization penalty to avoid over-fitting. Parameters $\lambda_{1}$ and $\lambda_{2}$ are to control the contribution of each part for collaborative decomposition.

Minimize the loss function above, is to find an optimal result for inferring the missing entries in tensor S with data correlation. But this loss function is not jointly convex to all the variables of A,R,H and T. In general, we cannot get closed-form solutions for the minimization. Therefore, we utilize a gradient descent technique [21] to get a local optimal solutions by minimizing the loss function iteratively. Finally, we recover S by $S_{rec}=A\times_{R}R\times_{H}H\times_{T}T$. Here, the multiplication symbol with subscript denotes tensor mode multiplication, e.g., $X=A\times_{R}R$ is $X_{n,j,k}=\sum_{i=1}^{d_{R}}A_{i,j,k}\times R_{n,i}$.

The mobility features of human crowd result in the uneven distribution of crowdsensing nodes, therefore it forms several hot/blank regions in urban area. For hot regions, there exist many crowdsensing nodes providing their measurements. As a result, different categories of sensory data co-exist in these regions, it is helpful for mining the signal correlation. On the other hand, there also exist some blank regions without any sensory data both for target signal and correlated signals. This means in such regions the target signal can’t be precisely recovered simply by collaborative tensor decomposition.

### 3.3. Correlated Time Slots Combination

As mentioned in Section 1, the frequency of crowdsensing nodes sending their measurements is much higher than that of environment changing, and the environment phenomenon will keep steady in a relative long period. To further supplement the missing items, we combine several continuous time slots together to generate a sensing image. For a specific crowdsensing node, correlated time slots combination is to make extension from time dimension. Due to the dynamic feature of crowdsensing nodes, in a period, these scattered crowdsensing nodes are more likely to form several sensing traces, therefore cover more regions.

**Figure 5:**
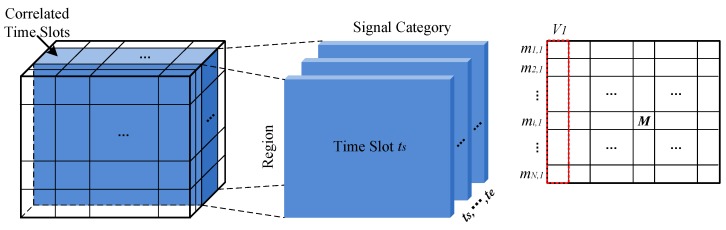
Correlated time slots combination.

Given a time period $T\in \left[t_{s},t_{e}\right]$, we extract the corresponding slices in tensor S and make an entry-wise combination (see Figure 5). Here, a specific extracted slice for time slot $t_{i}$ is a region-signal matrix. After that we formulate a matrix M (see the right part of Figure 5). An entry $m_{i,j}$ in matrix M, is computed by:

$$\begin{array}{c} m_{i,j}=\left\{\begin{array}{cc} \frac{\sum_{t_{s}\leq t\leq t_{e}}S\left(i,j,t\right)}{N}, & \;N\neq 0;\\ null, & \;N=0, \end{array}\right. \end{array}$$

where *N* denotes the number of sensory data with $S\left(i,j,t\right)\neq null$ in $\left[t_{s},t_{e}\right]$. The first column of M, denoted by $V_{1}={\left[m_{1,1},m_{2,1},\cdots ,m_{N,1}\right]}^{T}$, is the sensory data of target signal in *N* regions. By converting $V_{1}$ back into m×m matrix and using spacial interpolation method to further recover the missing entries, we get the optimized sensing image.

## 4. Numerical Simulation

In this section, we conduct numerical simulations to examine the promotion of our crowdsensing enhancement approach. Firstly, we illustrate the models we used to generate the mobile traces and environment signals. Then, we demonstrate an instance of the generated sensing images via enhanced crowdsensing approach. At last, we verify the promotion of our approach statistically.

### 4.1. Basic Models

*(1) Mobility model of crowdsensing nodes:* Existing works about human/vehicle mobility have summarized the following statistical features from real mobile traces: **F1** *heterogeneous bounded mobility areas*, **F2** *truncated power-law flights and pause-times*, **F3** *truncated power-law inter-contact times*, and **F4** *fractal waypoints*. In this paper, we utilize *Self-similar Least Action Walk* (SLAW) [11], that produces synthetic walk trajectories containing all above features, to simulate mobility traces of crowdsensing nodes. Parameter settings are summarized in Table 1. More details about SLAW model and the interpretation of these parameters can be found in [11].

**Figure 6:**
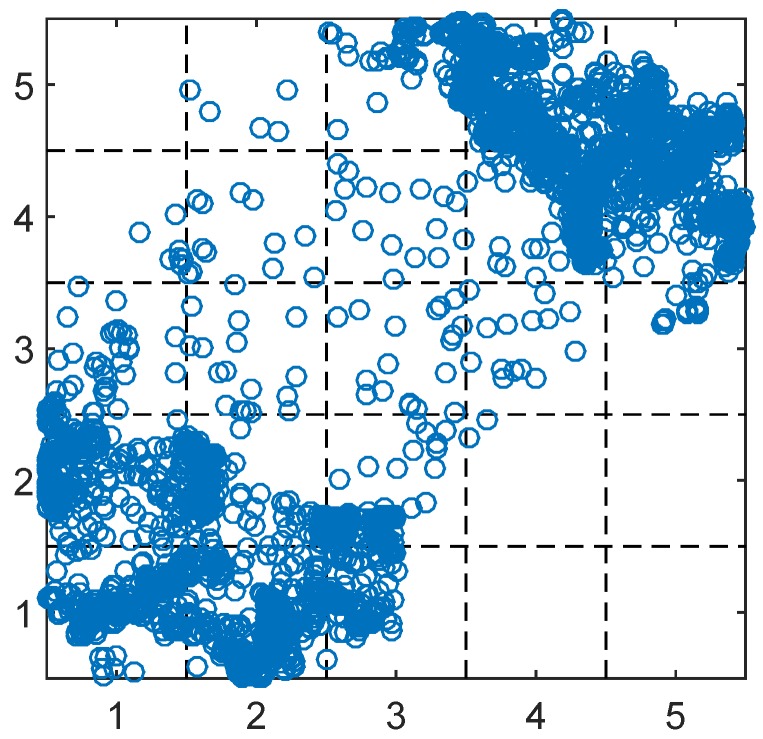
An instant of 6000 crowdsensing nodes generated by SLAW model.

After executing the SLAW model, we obtain all the positions of 6000 crowdsensing nodes for every minute over 10 h. Figure 6, illustrates the distribution of the crowdsensing nodes over the whole simulation region at a given instant. From Figure 6, we observe that some hot zones and blank zones exist simultaneously. This implies that the sensing ability of crowdsensing network for different urban regions may be very different. On the other hand, it is extremely complex to generate a sensing image of the whole city via all crowdsensing nodes in the city. Therefore, we divide the simulation region into 25 unit grids with $1\times 1\;{\mathrm{km}}^{2}$, and study the crowdsensing resolution for each grid.

**Figure 7:**
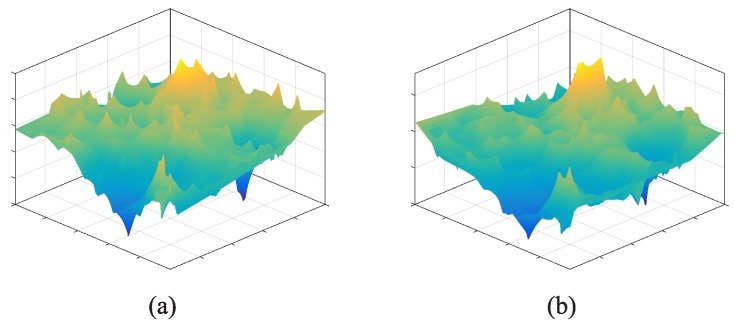
Correlated signals. (**a**) Linear correlated signal: $f_{1}\left(z_{i,j}\right)=0.95\times z_{i,j}+0.05+N\left(0,0.06\right)$; (**b**) Non-linear correlated signal: $f_{2}\left(z_{i,j}\right)=0.9\times z_{i,j}^{2}+0.9\times z_{i,j}+0.1+N\left(0,0.11\right)$.

*(2) Target and correlated signal models:* We utilize the 2D signal shown in Figure 2a as the ground truth of target signal. This is a signal of CO${}_{2}$ concentration which is generated by the sensory data from 100 CO${}_{2}$ sensor nodes in a square region (about 1 km${}^{2}$) of Wuxi City, China [24]. To generate the correlated signals, we utilized linear/non-linear transform function as follows:

$$\begin{array}{c} f_{1}\left(z_{i,j}\right)=az_{i,j}+b+e,\\ f_{2}\left(z_{i,j}\right)=az_{i,j}^{2}+bz_{i,j}+c+e, \end{array}$$

where *e*∼*N*$\left(0,\delta \right)$ is the additive noise. By adjusting the parameters of a,b,c and *δ*, we generate the linear/non-linear signals with 0.9 correlation (as shown in Figure 7).

### 4.2. Instance Illustration

**Figure 8:**
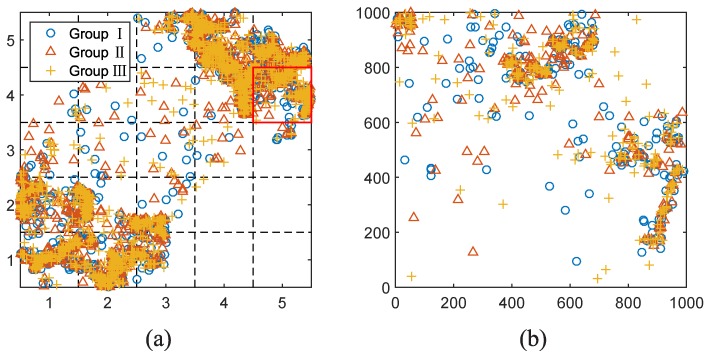
(**a**) Distribution of 6000 nodes (Groups I,II, and III ) over the whole 5×5 km${}^{2}$ region which is divided into 25 unit squares; (**b**) Distribution of crowdsensing nodes over the unit area indicated by the red box in (**a**).

We randomly divide the 6000 crowdsensing nodes into 3 groups (2000 nodes for each group) (see Figure 8a). Group I senses the target signal, Group II and III sense the correlated signals, respectively. Assuming that, the correlated time period of environment phenomenon is one hour. To estimate how the frequency of crowdsensing nodes sending their measurements affects crowdsensing promotion, we conduct two experiments by adjusting the sending interval as 10/20 min. Here, we extract the positions of sensing nodes from SLAW traces every 10/20 min to simulate different sending intervals. Therefore, for the time duration of one hour, we have 6/3 correlated time slots respectively. We name these two experiments by S6 and S3 respectively.

**Figure 9:**
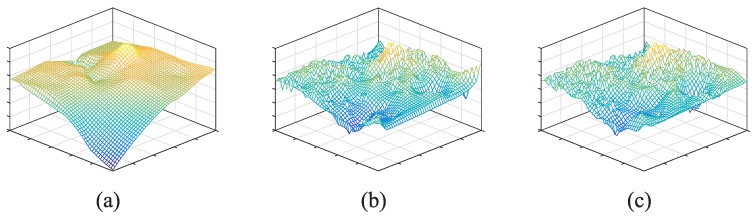
Recovered signals (generated sensing images). (**a**) Interpolation method; (**b**) Enhanced crowdsensing approach with 3 time slots combination; (**c**) Enhanced crowdsensing approach with 6 time slots combination.

As shown in Figure 9, for instance, we choose an unit grid (the red box in Figure 8a) to illustrate the recovered target signal after using enhanced crowdsensing approach. To make comparison, we also select a time slot in this one hour time period and generate a sensing image on the basis of interpolation method (as shown in Figure 9a).

**Figure 10:**
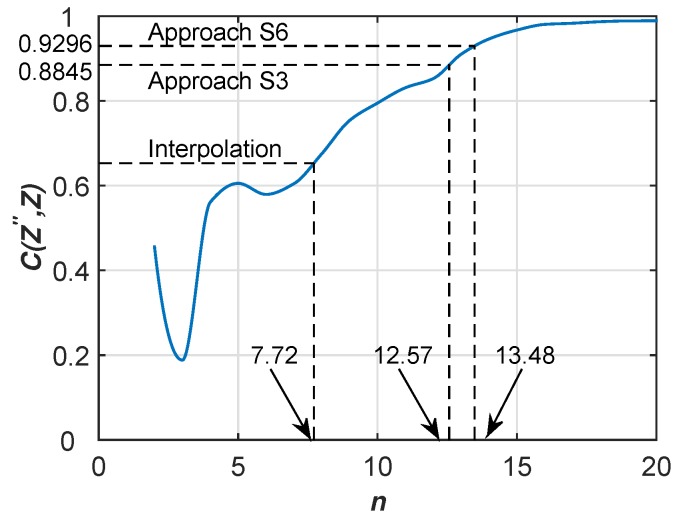
Correlation coefficients $C\left(Z^{\prime \prime },Z\right)$ against different values of *n*.

The qualities of Figure 9a–c are 0.653, 0.884 and 0.929, respectively. In this unit grid, the average number of crowdsensing nodes (Group I) is 247. Then, we use n×n gridded sampling points to generate sensing images and compute $C\left(Z^{\prime \prime },Z\right)$ against different values of *n* (see Figure 10). Let $C\left(Z^{\prime },Z\right)\approx C\left(Z^{\prime \prime },Z\right)$, we can easily acquire the corresponding $n_{l}$ as 7.72, 12.57 and 13.48 respectively.

### 4.3. Statistical Results

**Figure 11:**
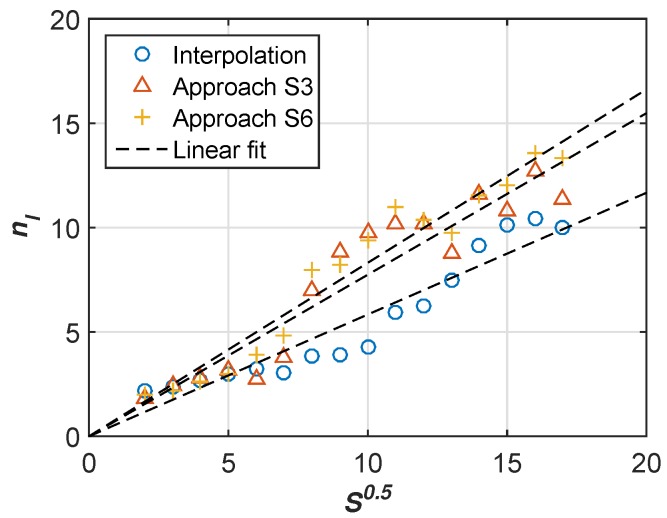
Relationship between crowdsensing resolution *r* and *s* crowdsensing nodes in an unit area which are generated by SLAW model. The x-axis denotes $\sqrt{s}$, and the y-axis denotes $n_{l}$, i.e., $\sqrt{r}$.

In this section, we analyze the crowdsensing promotion from statistical perspective. We count the number of Group I nodes for each grid at every time slots and calculate the average number of nodes for every hour. For each grid, we recover the target signal for every hour based on crowdsensing promotion approach with different sending intervals (10/20 min). Then we calculate the corresponding $n_{l}$ for each sensing image according to Equation (2). Therefore we have $25\;\mathrm{grids}\times 10\;h\times 1\;n_{l}$ values. We calculate the mean of $n_{l}$ which are with the same $\left\lfloor \sqrt{s}\right\rfloor$. Figure 11 plots the $n_{l}$ results against different values of $\sqrt{s}$. Moreover, we also plot the $n_{l}$ result based on interpolation method.

According to the linear regression results of simulations, we obtain the linear function $n_{l}=0.583\sqrt{s}$ for interpolation method. The slope *α* value of 0.583 is consistent with the result mentioned in paper [10]. We also get the linear functions: $n_{l}=0.774\sqrt{s}$ and $n_{l}=0.831\sqrt{s}$, for enhanced crowdsensing with S3 and S6 respectively. The higher value of *α* is, the higher resolution achieves for a given *s*. This statistical result validate that our enhanced crowdsensing approach outperforms the interpolation based method.

## 5. Case Study: Air Quality Monitoring in Beijing

The U-Air project [20] which is led by Microsoft Research aims to inference fine-grained air quality index (AQI) in Beijing with ubiquitous data. The researchers of U-Air open two data sources they used: locations of air monitoring stations in Beijing, and AQI (including the composition of air pollutants) of each station. In this section, we utilize a portion of these data to verify the effectiveness of our crowdsensing enhancement approach.

### 5.1. Data Correlation Analysis

**Figure 12:**
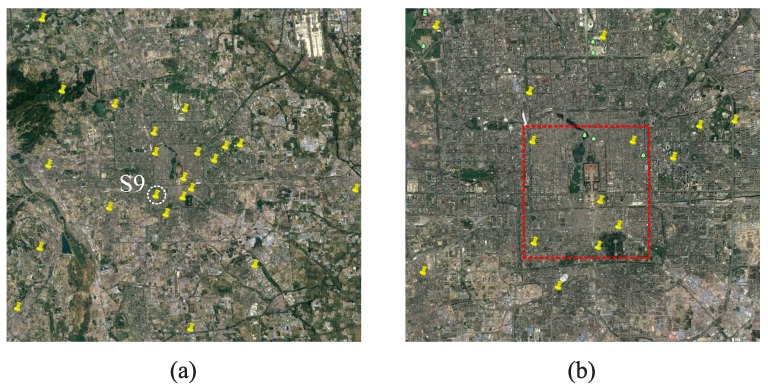
(**a**) Air quality monitoring stations in Beijing viewed from Google Earth; (**b**) The selected monitoring area within 2th ring road of Beijing.

In [20], researchers reveal that the concentration of air pollutants is notably influenced by meteorology (e.g., temperature, humidity) through analysing the reported information of monitoring stations. Figure 12a shows the locations of 22 monitoring stations in Beijing, and these stations report AQI every one hour. We extract the parameters of PM2.5, PM10 and air humidity index of monitoring station S9 within the time period from 21 to 27 April 2013, and quantize their correlation by correlation coefficients, denoted by C(·,·).

**Figure 13:**
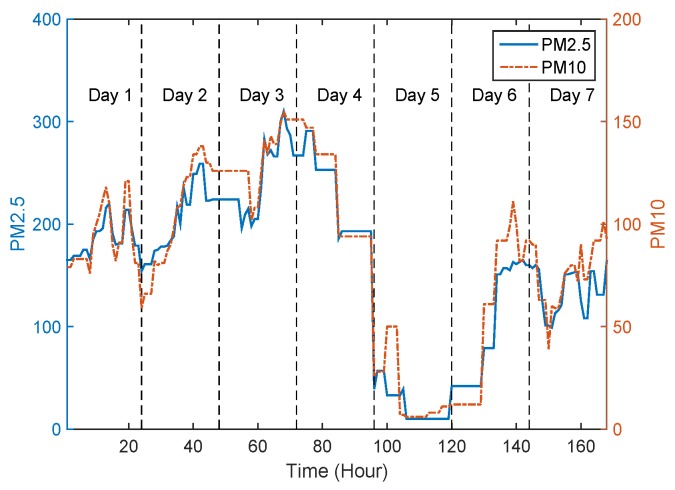
Variation tendency of PM2.5 and PM10 in monitoring station S9 during the time period of 21–27 April 2013.

**Figure 14:**
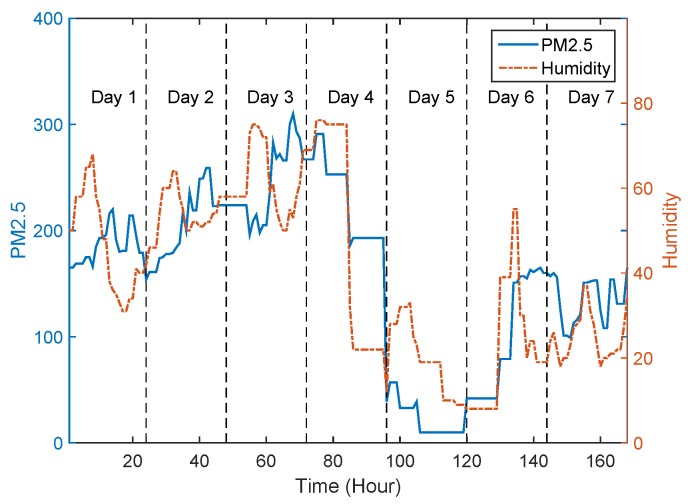
Variation tendency of PM2.5 and air humidity in monitoring station S9 during the time period of 21–27 April 2013.

Figure 13 and Figure 14 shows the variation tendency of PM2.5 versus PM10 and air humidity index, respectively. Both of the figures reflect clear correlation among PM2.5, PM10 and humidity. Specifically, the C(PM2.5,PM10), C(PM2.5,Humidity) are 0.91 and 0.75. Moreover, we also analyse the correlation among PM2.5, PM10 and air humidity index for the rest 21 monitoring stations. The statistical results are given in Table 2.

From Table 2, we observe that there are 20 monitoring stations with their C(PM2.5,PM10) above than 0.8, and 20 monitoring stations with their C(PM2.5,Humidity) above than 0.7, among all the 22 monitoring stations. This statistical result reveals strong correlation among PM2.5, PM10 and air humidity index.

### 5.2. Data Modelling

**Figure 15:**
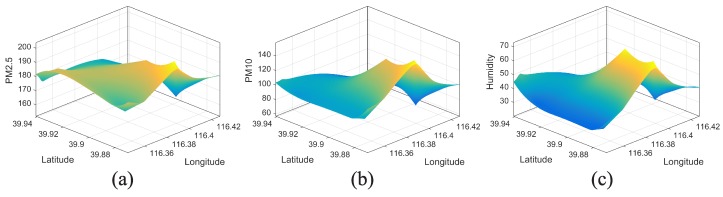
The constructed signals within the red box zones of Figure 12b. (**a**) Signal of PM2.5; (**b**) Signal of PM10; (**c**) Signal of air humidity.

Due to the lack of monitoring stations (Beijing only has 22 stations covering a 50×50 km${}^{2}$ land, i.e., 113 km${}^{2}$/per station [20]), it is not possible to acquire a fine-grained air pollutants distribution image for the whole urban area simply by spatial interpolation method. We choose the area within the second ring road of Beijing (see the red box zone in Figure 12b), which is the most concentrated area of monitoring station, as the target monitoring area. We map 3 categories of AQI (PM2.5, PM10 and air humidity index) onto the selected area according to the locations of their corresponding monitoring stations, and recover the signals by spatial interpolation method for time period around 12:00 at 24 April 2013 (see Figure 15).

From Figure 15, we observe similar distribution status among PM2.5, PM10 and air humidity. We take signal of PM2.5 as the target signal, while the others are correlated signals.

### 5.3. Comparison of Generated Sensing Images

**Figure 16:**
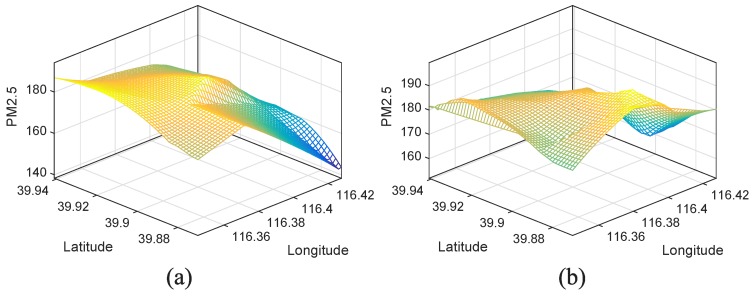
Recovered signals (sensing images). (**a**) The generated sensing image based on interpolation method; (**b**) The generated sensing image based on enhanced crowdsensing approach.

Microsoft Research shared a collection of taxicab GPS traces gathered in Beijing [25], and these traces are perceived with the statistical features of human mobility. In our crowdsensing network, we deem these ordinary taxicabs as the crowdsensing nodes and randomly select 100 different taxi traces within the target zones. Then, we extract an instant of the taxis’ GPS locations and sense the PM2.5 signal in Figure 15a. The generated sensing image by spatial interpolation method is shown in Figure 16a. Similar as mentioned in Section 4.2, we also extract the GPS locations of the selected traces for every 20 min to simulate the sensing cycle, then we have 3 time slots of sensing data within the one hour monitoring period. Moreover, we also select 100 traces to sense PM10 signal and 100 traces to sense air humidity signal. By conducting our crowdsensing enhancement approach, we acquire the optimized sensing image (see Figure 16b).

Compared with Figure 16a, the optimized sensing image of Figure 16b holds more details and the correlation coefficient between Figure 15a and Figure 16a is 0.58, while the correlation coefficient between Figure 15a and Figure 16b is 0.91. This result validates the utility of our method.

## 6. Conclusions

This paper studied the problem of how to promote the sensing ability of crowdsensing network for fine-grained environmental monitoring. In practice, the sensing ability of crowdsensing is limited by the number and space distribution of crowdsensing participants. To enhance the ability of crowdsensing, we proposed a multi-source data driven approach and then utilized a novel metric called “crowdsensing resolution” to quantize the enhancement. The kernel of our enhanced crowdsensing approach, is to leverage temporal and sensory correlated data to help with the recovery of sensing image in blank zones. By improving the sensing ability for blank zones, the crowdsensing network could generate sensing images more precisely, i.e., achieve a higher crowdsensing resolution. More specifically, we built a tensor to model the sensory data and summarized a signal correlation matrix to quantize the correlation between different categories of sensory data. By conducting collaborative tensor decomposition and correlated time slots combination, we presented the optimized sensing images. The numerical simulations and a real dataset based case study verified the resolution promotion of our enhanced crowdsensing beyond traditional interpolation-based approach.

## Figures and Tables

**Table 1:** Parameter settings of SLAW model.

Parameter	Value
Distance alpha	3
Number of mobile nodes	6000
Simulation area	$5000\times 5000\;m^{2}$
Number of waypoints	6000
Hurst parameter	0.75
Time duration	10 h
Clustering range	100 m
Levy exponent for pause time	1
Minimum/maximum pause time	30 s/1800 s

**Table 2:** Statistical result of correlation analysis.

Value Ranges of C(·,·)	Number of Monitoring Stations	
	C(PM2.5,PM10)	C(PM2.5,Humidity)
C(·,·)∈[0.9,1.0)	13	0
C(·,·)∈[0.8,0.9)	7	1
C(·,·)∈[0.7,0.8)	1	19
C(·,·)∈[0.5,0.7)	1	2
